# Supplementary material for: Machine Learning Accelerates Crystallization for Structure Determination
Source: Angew Chem Int Ed Engl. 2026 May 4;65(25):e1218503. doi: 10.1002/anie.1218503 (PMC13266958; doi:10.1002/anie.1218503)

checkCIF/PLATON report

Structure factors have been supplied for datablock(s) lcz004

THIS REPORT IS FOR GUIDANCE ONLY. IF USED AS PART OF A REVIEW PROCEDURE FOR PUBLICATION, IT SHOULD NOT REPLACE THE EXPERTISE OF AN EXPERIENCED CRYSTALLOGRAPHIC REFEREE.

No syntax errors found. CIF dictionary Interpreting this report

Datablock: lcz004

```
Bond precision:    C-C = 0.0155 A           Wavelength=1.54184

Cell:              a=8.6774(2)      b=9.1450(2)        c=41.2028(6)
                   alpha=90         beta=93.794(2)     gamma=90
Temperature:       100 K
```

	Calculated	Reported
Volume	3262.48(11)	3262.48(11)
Space group	P 21/n	P 1 21/n 1
Hall group	-P 2yn	-P 2yn
Moiety formula	C24 H13 Ag3 F18 N6 O4	C15 H3 Ag3 F18 N6, C9 H10 O4
Sum formula	C24 H13 Ag3 F18 N6 O4	C24 H13 Ag3 F18 N6 O4
Mr	1115.02	1115.01
Dx,g cm-3	2.270	2.270
Z	4	4
Mu (mm-1)	15.692	15.692
F000	2136.0	2136.0
F000’	2146.55	
h,k,lmax	10,11,52	10,11,52
Nref	6934	6640
Tmin,Tmax	0.190,0.208	0.563,1.000
Tmin’	0.035	

```
Correction method= # Reported T Limits: Tmin=0.563 Tmax=1.000
AbsCorr = MULTI-SCAN
```

Data completeness= 0.958 Theta(max)= 77.730

R(reflections)= 0.0715(5764) wR2(reflections)= 0.1746(6640)

S = 1.092 Npar= 499

The following ALERTS were generated. Each ALERT has the format
test-name_ALERT_alert-type_alert-level.
Click on the hyperlinks for more details of the test.

Alert level C

```
CRYSC01_ALERT_1_C The word below has not been recognised as a standard
            identifier.
            dull
PLAT042_ALERT_1_C Calc. and Reported MoietyFormula Strings  Differ     Please Check
              Calc: C24 H13 Ag3 F18 N6 O4
              Rep.: C15 H3 Ag3 F18 N6, C9 H10 O4
PLAT213_ALERT_2_C Atom F00F           has ADP max/min Ratio .....        3.7 prolat
PLAT213_ALERT_2_C Atom F00L           has ADP max/min Ratio .....        3.4 prolat
PLAT213_ALERT_2_C Atom F00N           has ADP max/min Ratio .....        3.2 prolat
PLAT213_ALERT_2_C Atom F00X           has ADP max/min Ratio .....        3.4 prolat
PLAT213_ALERT_2_C Atom F010           has ADP max/min Ratio .....        3.5 prolat
PLAT213_ALERT_2_C Atom F01E           has ADP max/min Ratio .....        3.6 prolat
PLAT220_ALERT_2_C NonSolvent   Resd 1  F   Ueq(max)/Ueq(min) Range       3.5 Ratio
PLAT234_ALERT_4_C Large Hirshfeld Difference F01J     --C01H     .      0.20 Ang.
PLAT234_ALERT_4_C Large Hirshfeld Difference C00Z     --C015     .      0.16 Ang.
PLAT234_ALERT_4_C Large Hirshfeld Difference C015     --C016     .      0.18 Ang.
PLAT241_ALERT_2_C High   'MainMol' Ueq as Compared to Neighbors of      O00Q Check
PLAT342_ALERT_3_C Low Bond Precision on  C-C Bonds ..............    0.01547 Ang.
PLAT369_ALERT_2_C Long   C(sp2)-C(sp2) Bond  C015     - C016     .      1.54 Ang.
PLAT434_ALERT_2_C Short Inter HL..HL Contact F01J     ..F01J     .      2.50 Ang.
                                                1-x,1-y,1-z  =      3_666 Check
PLAT906_ALERT_3_C Large K Value in the Analysis of Variance ......     11.831 Check
PLAT906_ALERT_3_C Large K Value in the Analysis of Variance ......      2.239 Check
PLAT911_ALERT_3_C Missing FCF Refl Between Thmin & STh/L=    0.600         10 Report
               -1  0  1,  -4 10  1,  -4  9  2, -10  2  4,  -7  8  6,   9  0 11,
                2  3 14,  -6  8 16,  -7  7 17,  -7  3 36,
PLAT971_ALERT_2_C Check Calcd Resid. Dens.  1.31Ang From F01E           2.16 eA-3
PLAT971_ALERT_2_C Check Calcd Resid. Dens.  0.92Ang From Ag03           2.10 eA-3
PLAT971_ALERT_2_C Check Calcd Resid. Dens.  0.99Ang From Ag01           1.79 eA-3
PLAT971_ALERT_2_C Check Calcd Resid. Dens.  1.27Ang From O00Q           1.75 eA-3
PLAT971_ALERT_2_C Check Calcd Resid. Dens.  1.10Ang From F01J           1.69 eA-3
PLAT971_ALERT_2_C Check Calcd Resid. Dens.  0.96Ang From Ag02           1.68 eA-3
PLAT971_ALERT_2_C Check Calcd Resid. Dens.  0.99Ang From F01J           1.62 eA-3
PLAT971_ALERT_2_C Check Calcd Resid. Dens.  1.03Ang From Ag03           1.55 eA-3
PLAT971_ALERT_2_C Check Calcd Resid. Dens.  0.99Ang From Ag02           1.53 eA-3
PLAT972_ALERT_2_C Check Calcd Resid. Dens.  0.44Ang From F01I          -1.63 eA-3
PLAT972_ALERT_2_C Check Calcd Resid. Dens.  0.95Ang From Ag03          -1.56 eA-3
PLAT972_ALERT_2_C Check Calcd Resid. Dens.  0.83Ang From Ag01          -1.55 eA-3
PLAT977_ALERT_2_C Check Negative Difference Density on H00Y      .     -0.32 eA-3
PLAT977_ALERT_2_C Check Negative Difference Density on H013      .     -0.45 eA-3
PLAT977_ALERT_2_C Check Negative Difference Density on H01G      .     -0.31 eA-3
```

Alert level G

```
PLAT003_ALERT_2_G Number of Uiso or U(i,j)  Restrained non-H-Atoms          3 Report
PLAT004_ALERT_5_G Polymeric Structure Found with Maximum Dimension          1 Info
PLAT083_ALERT_2_G SHELXL Second Parameter in WGHT  Unusually Large      81.92 Why ?
PLAT186_ALERT_4_G The CIF-Embedded .res File Contains ISOR Records          1 Report
PLAT242_ALERT_2_G Low    'MainMol' Ueq as Compared to Neighbors of       C00W Check
PLAT242_ALERT_2_G Low    'MainMol' Ueq as Compared to Neighbors of       C011 Check
PLAT242_ALERT_2_G Low    'MainMol' Ueq as Compared to Neighbors of       C017 Check
PLAT242_ALERT_2_G Low    'MainMol' Ueq as Compared to Neighbors of       C018 Check
PLAT242_ALERT_2_G Low    'MainMol' Ueq as Compared to Neighbors of       C01F Check
PLAT242_ALERT_2_G Low    'MainMol' Ueq as Compared to Neighbors of       C01H Check
PLAT333_ALERT_2_G Large Aver C6-Ring C-C Dist C00Z     -C01B     .       1.43 Ang.
PLAT335_ALERT_2_G Check Large C6 Ring C-C Range C00Z     -C01B           0.24 Ang.
PLAT434_ALERT_2_G Short Inter HL..HL Contact F00X     ..F00X     .       2.83 Ang.
                                                 2-x,-y,1-z  =      3_756 Check
PLAT720_ALERT_4_G Number of Unusual/Non-Standard Labels ..........         68 Note
              Ag01    Ag02    Ag03    N004    F005    N006    F007    F008
              N009    O00A    N00B    N00C    F00D    F00E    F00F    F00G
              F00H    N00I    F00J    O00K    F00L    O00M    F00N    C00O
              C00P    O00Q    F00R    F00S    C00T    H00T    C00U    C00V
              C00W    F00X    C00Y    H00Y    C00Z    F010    C011    C012
              C013    H013    C014    C015    C016    C017    C018    C019
              C01A    H01A    H01B    H01C    C01B    H01D    C01C    H01E
              H01F    H01G    C01D    F01E    C01F    C01G    H01H    H01I
              H01J    C01H    F01I    F01J
PLAT860_ALERT_3_G Number of Least-Squares Restraints .............         18 Note
PLAT883_ALERT_1_G Absent Datum for _atom_sites_solution_primary ..     Please Do !
PLAT910_ALERT_3_G Missing FCF Reflection(s) Below Theta(Min)[Deg]=       4.95 Note
                0  0  2,   0  0  4,
PLAT912_ALERT_4_G Missing # of FCF Reflections Above STh/L=  0.600        281 Note
PLAT933_ALERT_2_G Number of HKL-OMIT Records in Embedded .res File          3 Note
               -6  8 16,  -4  9  2, -10  2  4,
PLAT941_ALERT_3_G Average HKL Measurement Multiplicity ...........        2.8 Low
PLAT969_ALERT_5_G The 'Henn et al.' R-Factor-gap value ...........      3.770 Note
              Predicted wR2: Based on SigI**2  4.63 or SHELX Weight 15.98
PLAT978_ALERT_2_G Number C-C Bonds with Positive Residual Density.          0 Info
```

```
   0 ALERT level A = Most likely a serious problem - resolve or explain
   0 ALERT level B = A potentially serious problem, consider carefully
  34 ALERT level C = Check. Ensure it is not caused by an omission or oversight
  22 ALERT level G = General information/check it is not something unexpected

   3 ALERT type 1 CIF construction/syntax error, inconsistent or missing data
  38 ALERT type 2 Indicator that the structure model may be wrong or deficient
   7 ALERT type 3 Indicator that the structure quality may be low
   6 ALERT type 4 Improvement, methodology, query or suggestion
   2 ALERT type 5 Informative message, check
```

Validation response form

Please find below a validation response form (VRF) that can be filled in and pasted into your CIF.

```
# start Validation Reply Form
_vrf_CRYSC01_lcz004
;
PROBLEM: The word below has not been recognised as a standard
RESPONSE: ...
;
_vrf_PLAT042_lcz004
;
PROBLEM: Calc. and Reported MoietyFormula Strings  Differ     Please Check
RESPONSE: ...
;
_vrf_PLAT213_lcz004
;
PROBLEM: Atom F00F            has ADP max/min Ratio .....        3.7 prolat
RESPONSE: ...
;
_vrf_PLAT220_lcz004
;
PROBLEM: NonSolvent   Resd 1  F   Ueq(max)/Ueq(min) Range        3.5 Ratio
RESPONSE: ...
;
_vrf_PLAT234_lcz004
;
PROBLEM: Large Hirshfeld Difference F01J     --C01H     .       0.20 Ang.
RESPONSE: ...
;
_vrf_PLAT241_lcz004
;
PROBLEM: High   'MainMol' Ueq as Compared to Neighbors of       O00Q Check
RESPONSE: ...
;
_vrf_PLAT342_lcz004
;
PROBLEM: Low Bond Precision on  C-C Bonds ..............    0.01547 Ang.
RESPONSE: ...
;
_vrf_PLAT369_lcz004
;
PROBLEM: Long   C(sp2)-C(sp2) Bond  C015     - C016     .       1.54 Ang.
RESPONSE: ...
;
_vrf_PLAT434_lcz004
;
PROBLEM: Short Inter HL..HL Contact F01J     ..F01J     .       2.50 Ang.
RESPONSE: ...
;
_vrf_PLAT906_lcz004
;
PROBLEM: Large K Value in the Analysis of Variance ......     11.831 Check
RESPONSE: ...
;
_vrf_PLAT911_lcz004
;
PROBLEM: Missing FCF Refl Between Thmin & STh/L=    0.600         10 Report
RESPONSE: ...
;
_vrf_PLAT971_lcz004
```

```
;
PROBLEM: Check Calcd Resid. Dens.  1.31Ang From F01E            2.16 eA-3
RESPONSE: ...
;
_vrf_PLAT972_lcz004
;
PROBLEM: Check Calcd Resid. Dens.  0.44Ang From F01I           -1.63 eA-3
RESPONSE: ...
;
_vrf_PLAT977_lcz004
;
PROBLEM: Check Negative Difference Density on H00Y      .      -0.32 eA-3
RESPONSE: ...
;
# end Validation Reply Form
```

It is advisable to attempt to resolve as many as possible of the alerts in all categories. Often the minor alerts point to easily fixed oversights, errors and omissions in your CIF or refinement strategy, so attention to these fine details can be worthwhile. It is up to the individual to critically assess their own results and, if necessary, seek expert advice.

PLATON version of 17/09/2025; check.def file version of 17/09/2025

---

duplicate check

No duplication found

Datablock lcz004 - ellipsoid plot

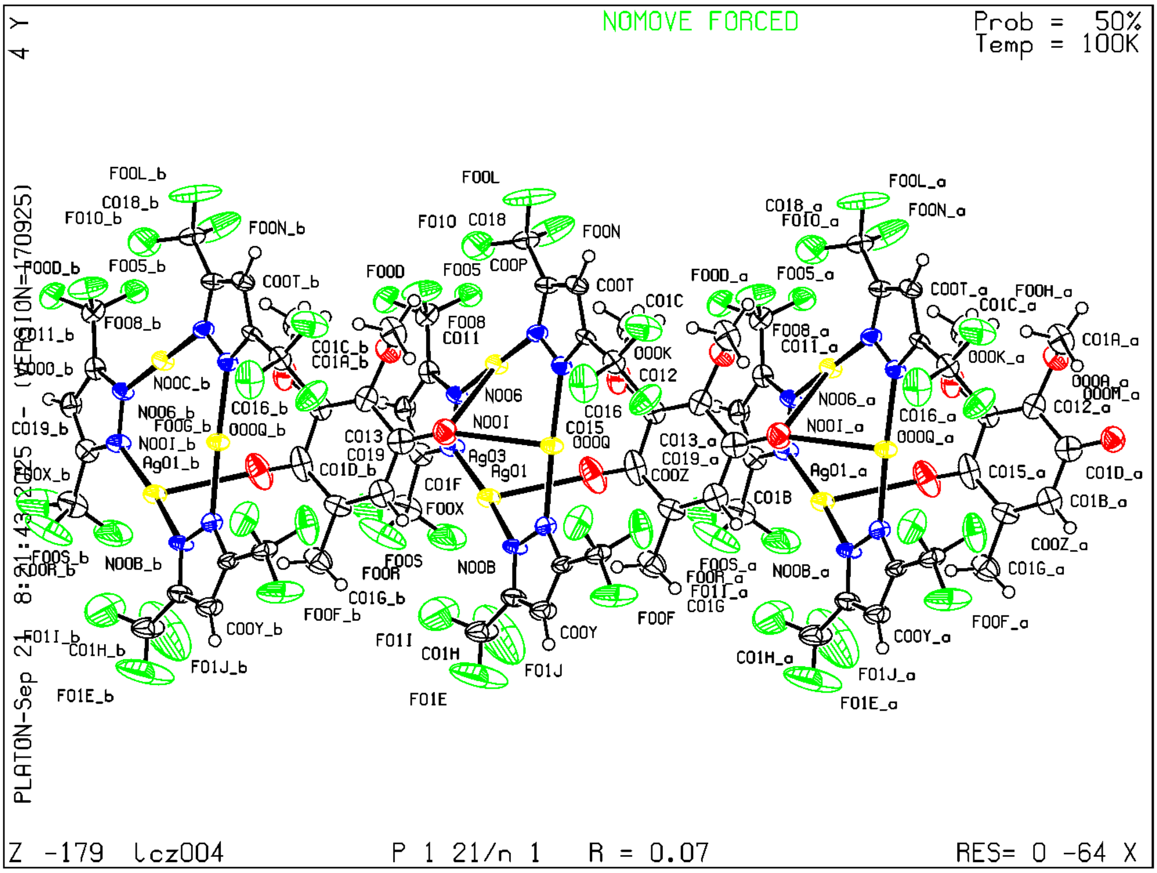

Supplement: Supplementary file 2 — Supporting File 2: anie72462‐sup‐0002‐DataFile.zip. [file ANIE-65-e1218503-s002.zip › supplementary_materials_crystallographic_data submit.zip!/cif_and_checkcif(1-114)/Ag3Pz3_4/Ag3Pz3_4_checkcif.pdf]
